# Composting and its application in bioremediation of organic contaminants

**DOI:** 10.1080/21655979.2021.2017624

**Published:** 2022-01-09

**Authors:** Chitsan Lin, Nicholas Kiprotich Cheruiyot, Xuan-Thanh Bui, Huu Hao Ngo

**Affiliations:** a Maritime Science and Technology, College of Maritime, National Kaohsiung University of Science and Technology, Kaohsiung, Taiwan (R.O.C.); b Department of Marine Environmental Engineering, National Kaohsiung University of Science and Technology, Kaohsiung, Taiwan (R.O.C.); c Key Laboratory of Advanced Waste Treatment Technology, Ho Chi Minh City University of Technology (HCMUT), Vietnam National University Ho Chi Minh (VNU-HCM), Ho Chi Minh City, Vietnam; d Faculty of Environment & Natural Resources, Ho Chi Minh City University of Technology (Hcmut), Ho Chi Minh City, Vietnam; e Centre for Technology in Water and Wastewater, School of Civil and Environmental Engineering, University of Technology Sydney, Sydney, Australia

**Keywords:** Aerobic biodegradation, bioaugmentation, bio-stimulation, green technology, microbial community, recalcitrant organic contaminants

## Abstract

This review investigates the findings of the most up-to-date literature on bioremediation via composting technology. Studies on bioremediation via composting began during the 1990s and have exponentially increased over the years. A total of 655 articles have been published since then, with 40% published in the last six years. The robustness, low cost, and easy operation of composting technology make it an attractive bioremediation strategy for organic contaminants prevalent in soils and sediment. Successful pilot-and large-scale bioremediation of organic contaminants, e.g., total petroleum hydrocarbons, plasticizers, and persistent organic pollutants (POPs) by composting, has been documented in the literature. For example, composting could remediate >90% diesel with concentrations as high as 26,315 mg kg^−a^ of initial composting material after 24 days. Composting has unique advantages over traditional single- and multi-strain bioaugmentation approaches, including a diverse microbial community, ease of operation, and the ability to handle higher concentrations. Bioremediation via composting depends on the diverse microbial community; thus, key parameters, including nutrients (C/N ratio = 25–30), moisture (55–65%), and oxygen content (O_2_ > 10%) should be optimized for successful bioremediation. This review will provide bioremediation and composting researchers with the most recent finding in the field and stimulate new research ideas.

## 1. Introduction

Composting is a self-heating biological process that has been used for centuries as an organic waste management solution. Apart from managing organic waste, the composting product can be used as a soil amendment and organic fertilizer. Composting research has made substantial advances over the years, especially on shortening the composting process and improving compost quality. The research has been aided by the knowledge of parameters affecting the composting process, including initial particle size, nutrients, oxygen content, moisture content, pH, and temperature. In addition, because composting can biodegrade organic products, researchers have been interested in using this technology to treat recalcitrant organic contaminants, including polycyclic aromatic hydrocarbons (PAHs) [1–3], total petroleum hydrocarbons (TPHs) [4], diesel [5], phthalate-based plasticizers [6,7], organochlorine pesticides [8], polychlorinated dibenzo-p-dioxins and furans (PCDD/Fs) [9,10], and polychlorinated biphenyls (PCBs) [11].

The diverse microbial communities present in composting materials are responsible for the biodegradation of recalcitrant organic contaminants. This degradation process could take the form of either complete mineralization/metabolism, co-metabolism, or nonspecific extracellular oxidation. Several studies have identified species that can mineralize these contaminants. For instance, *Acinetobacter lwoffii, Bacillus subtilis* and *Raoultella ornithinolytica* can degrade crude oil [12]. Moreover, high temperatures during the composting (thermophilic phase) also enhance degradation by making the contaminants less viscous and more bioavailable. Biosurfactants, e.g., rhamnolipids produced by certain microbial species during composting, also enhance biodegradation by solubilizing the organic contaminants.

Therefore, this review is aimed at presenting, reviewing, and discussing the recent bioremediation via composting literature. Past review papers, published in the past ten years, have only focused on specific organic contaminants, including TPH [13], PAHs [14–17], and pesticides [16,18]. This review will include all the organic contaminants that have been reported to degrade during composting. Because composting is a biological process and is influenced by some key physicochemical parameters, this review also includes an overview of the composting process and these parameters to enable the readers to understand that the key to effective bioremediation via composting lies in the optimization of the parameters. In addition, the composting studies are compared to commonly used single- and multi-strain bioremediation approaches to gauge the competitiveness of this technology. We conclude the review by offering some future perspectives in this research field that we believe would stimulate research ideas that are equally beneficial and interesting.

## 2. Overview of organic waste composting process

### 2.1. Basics of the composting process

The composting process has been neatly categorized into four phases: *mesophilic, thermophilic, cooling*, and *maturation*. These phases have different temperature, oxygen demand, microbial community structure, stability, carbon content, nitrogen content, and pH profiles. After the initial composting mixture has been prepared, the mesophilic phase commences. The microbes utilize readily degradable organic matter as a nutrient source. As a result, the temperature rises above the ambient temperature after hours, or even a few days, depending on the composting scale, initial material, and composting conditions. If the compost mixture has soluble organic compounds such as sugars, organic acids may be produced during the fermentation of these compounds, resulting in a pH drop into the acidic range. However, the pH will not stay in this range for long due to the further decomposition of organic acids, volatilization, and the production of NH_3_. This phase lasts until the temperature reaches 55°C, ushering in the thermophilic phase. This stage has the highest temperature during the composting process.

Since temperature is an indicator of microbial activity, the initial stage of the thermophilic phase is considered the period with the highest activity. Mesophilic microbes are temperature sensitive and deactivated during the thermophilic phase, while thermophilic microbes populate the microbial community. Less biodegradable and complex organic substances like cellulose and hemicellulose start biodegrading during this phase. Ammonia produced from the degradation of nitrogen-containing organic matter causes an increase in pH [19]. The high temperature in this period also destroys most human and animal pathogens such as *Escherichia coli* and *Salmonella sp*. Microbial activity slows down as nutrient sources deplete, causing a decrease in temperature and the beginning of the curing phase, which consists of the cooling and the maturation phase. The temperature during the cooling phase is similar to the mesophilic phase, and mesophilic organisms thrive during this stage. The available nutrient source comprises complex organic materials that are lignocellulosic. Macrofungi, which consume these complex materials, are usually observed in the compost, while the pH remains alkaline but drops slightly, approaching the neutral range. The cooling phase generally takes several weeks and can easily be mistaken for the maturation phase, the last stage of the composting process when the compost is stable and mature. The end product is a humus-like substance with an earthy smell. At this period, the compost temperature is similar to the ambient temperature, and the pH is neutral or slightly alkaline. Several indicators, including the germination index and soluble C/N ratio, are used to determine the maturity and stability of the compost [20].

### 2.2. Key parameters for effective composting

**Figure 1.**
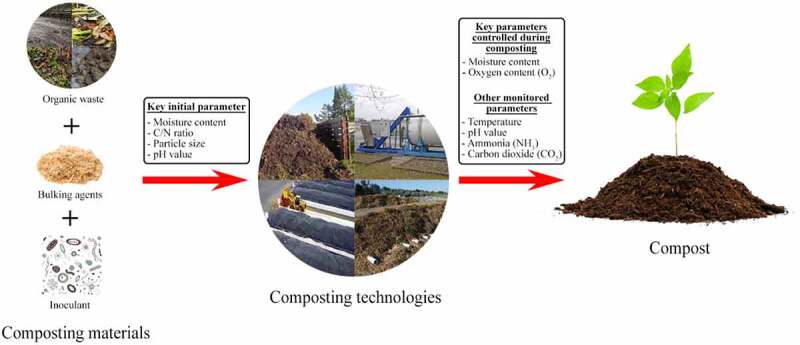
Overview of the key parameters during the composting process.

Composting, like other biological processes, is affected by nutrient availability and environmental conditions. This subsection will discuss the key parameters that influence the process and also include the optimal conditions for effective composting where necessary. Figure 1 summarizes these parameters and separates them into those initially adjusted and those monitored and/or controlled throughout the composting process. Moisture content, C/N ratio, particle size, and in some cases pH, are initially controlled to provide microorganisms with a suitable environment for thriving. Throughout the composting process, parameters, including oxygen and moisture content, that influence microbial activity during the four stages of the process, are monitored and controlled.

#### 2.2.1. Initial compost materials and nutrient balance

The microorganisms in the compost require macronutrients such as carbon, nitrogen, phosphorus, and potassium, and micronutrients, including essential metals and minerals. The source of these nutrients is the substrate or feedstock available for these microorganisms. Another aspect to consider is how readily the microorganisms can break down the substrates. For example, recalcitrant substances like cellulose and lignin would take longer to break down compared to fructose. Consequently, although nutrients might be present in a substrate, they must be in a form that the microbes can utilize. Additionally, the decomposition depends on the enzymatic composition of individual microorganisms, strongly suggesting that some microbes can break down specific substrates while others might only break down the intermediate products.

In composting, carbon and nitrogen contents of composting materials are described as the main nutritional characteristic of the substrate. Carbon is used mainly as an energy source, while nitrogen is necessary for cell growth and function. The C/N ratio is used in composting to assess whether the microbes have sufficient nutrients. Table 1 shows the nitrogen content and C/N ratio of commonly used composting materials. Generally, animal manure and sewage sludge are usually rich in nitrogen from urine and have lower C/N ratios, while lignocellulosic materials such as wheat and rice straw have more carbon and, therefore, a high C/N ratio. The consensus among most researchers is that an initial C/N ratio of 25–30 is ideal for the composting process. However, since the range assumes complete carbon mineralization, lower C/N ratios of up to 14 have been shown to work well [21].

**Table 1.** Commonly used compost materials and their nitrogen and C/N ratio reported in literature

Compost materials	Nitrogen	C/N ratio	Ref.
Cow manure	2.3–2.6	13.8–15.8	^[92,93]^
Chicken manure	2.6–7.0	6.1–8.9	^[94,95]^
Pig manure	2.5–3.0	16.8–17.2	^[96,97]^
Sheep manure	2.1–2.8	17–17.1	^[98,99]^
Sewage sludge	1.7–4.5	11.3–11.5	^[95,100]^
Sawdust	0.5–1.8	140–250	^[100,101]^
Rice straws	0.25–0.54	36.4–71.7	^[92,93]^
Wheat straw	0.8–0.9	69.6–77.5	^[102,103]^

Lower ratios in excess of the requirements of the microbial population would lead to nitrogen loss as volatilized ammonia [22], leading to malodor pollution. On the other hand, higher ratios lead to longer composting processes due to limited nitrogen resources. Therefore, the initial C/N ratio is usually adjusted before composting. Materials such as sucrose [23–25], glucose [26], spent mushroom [24], and cellulose [26] have been used to increase the C/N ratio of the compost mixture and reduce ammonia loss. For instance, Meng, et al. [26] showed that 4% addition of sucrose to sewage sludge increased the C/N ratio from 8.06 to 9.56 and decreased nitrogen loss by 46.3%. Dry leaves and straw, which are common bulking agents, have a very high C/N ratio and, when added to the compost mixture, increase the initial C/N ratio. However, the types of carbon, for instance, lignin in these bulking agents, are complex and difficult to degrade. Another strategy that has been used to inhibit nutrient loss, and most specifically nitrogen loss in the form of ammonia, comprises biochar and other adsorbents [27,28]. These substances have high surface areas and adsorb ammonia preventing volatilization. High C/N ratios have been adjusted using ammonium fertilizers to decrease the C/N ratio, especially for some commercial-scale composting facilities [29]. However, this adds to the operational cost.

#### 2.2.2. Initial particle size

The particle size of the initial composting materials is important in two aspects. First, the size of the particle determines the surface area on which microbes can consume. Second, the particle size dictates how homogenous the initial materials mix. Smaller particles have larger surface areas which would allow for effective degradation. They also improve the homogenous mixing of the initial materials. However, small particles might also inhibit air and water penetration within the mixture leading to anaerobic zones. Conversely, larger particle sizes can lead to excessive ventilation, diminished water holding capacity, and slower degradation [30]. There is no consensus about the best possible particle size for composting. Studies have used different particle sizes in their investigations, for instance, ≤ 1 cm in food waste composting [9,20,31] and 1.5–3.0 cm in composting of cattle, chicken, kitchen, and municipal solid waste [21]. Some researchers have studied the effect of particle size of the bulking agents on the composting process. For example, He, et al. [32] found that granular biochar reduced methane emissions during pig manure and wheat straw composting by 22.2%, while powdered biochar increased emissions by 56.8%. This observation implies that anaerobic conditions occurred more frequently in the treatments with powdered biochar since methane is produced by methanogens which are anaerobic microorganisms. Bulking agents are supposed to give compost structural integrity, and the powdered biochar was too fine, resulting in poor air and water penetration. For the engineering and financial aspects, the grinding/cutting cost versus the additional benefit should be weighed when choosing the preferred particle size.

#### 2.2.3. Moisture content

Moisture content is an important parameter in the composting process because microorganisms need adequate moisture to survive. Water is necessary for the transport of nutrients, making them accessible to microbes. Moisture influences air penetration, nutrients, oxygen uptake, and temperature. Higher moisture (usually >70%) content during the composting process forms waterlogs that lead to anaerobic conditions. Lower moisture content (usually <40%) could cause early dehydration during composting, hindering the biological process. However, the optimal moisture content depends on the feedstock’s physical characteristics, including the particle size and water-holding capacity, but a range of 55–65% has been utilized by most composting studies treating various types of organic materials [13,31–33]. The moisture content will also vary throughout the composting process depending on the temperature and aeration. For this reason, the moisture content is continuously adjusted, especially during the thermophilic phase.

#### 2.2.4. Oxygen content

The aerobic microorganisms in compost require oxygen for respiration, so oxygen supply is crucial during composting. It is important that the microorganisms are provided with adequate oxygen to maintain their metabolic activities throughout composting. The oxygen content of > 10% in the compost gas throughout composting is recommended [13]. Oxygen is supplied either through turning the compost manually or mechanically or with the aid of an aeration pump using positive or negative pressure depending on the size of the compost and resources. Among all other parameters mentioned in this review, aeration is the most influenced by the technology. In addition to supplying oxygen, aeration influences temperature and moisture during composting. Inadequate aeration leads to anaerobic conditions, while too high might dry out the compost and inhibit the composting process [34]. Furthermore, since oxygen demand is proportional to microbial activities, aeration should be the highest during the thermophilic phase and the lowest during the curing phases.

Besides the composting scale, the desired aeration rate will depend on the characteristics of the composting materials, including particle size and moisture content [35,36]. Specifically, the particle size of the bulking agents, which provide the structural integrity of the compost mixture, will influence the oxygen supply in the compost. Therefore, a bulking agent that provides adequate voids that allow oxygen penetration throughout the compost is recommended. As Cao, et al. [35] showed, powdered bulking agents increased methane emissions. Water and air compete for these interstitial voids, and therefore high moisture implies that the voids are occupied with water instead of air. In their extensive literature review, Tran, et al. [13] concluded that an optimal aeration rate of 1–2 L kg _dry wt._^−1^ min^−1^ could meet the aeration requirements for a successful pilot-scale composting process. However, it is not possible to maintain complete aerobic conditions during composting, especially for large-scale composting. Therefore, the goal is to maximize aeration within the constraints of financial feasibility.

#### 2.2.5. Temperature

Composting is a self-heating biological process, and the heat is a product of aerobic microbial degradation of organic matter. The produced heat influences moisture and microbial community structure [37]. High temperatures have been shown to dry out the compost and inhibit the composting process. In addition, the microbial diversity decreases in high temperatures, and only thermophiles, e.g., *Thermus* genus, survive and thrive under such conditions. For instance, Yu, et al. [38] observed that the Shannon index, a measure of microbial diversity, dropped from 7.86 at day 0 to 4.03 at day 3 when the temperature reached 93.4°C, during hyperthermophilic composting. However, this is not necessarily a bad thing. Hyperthermophilic composting is garnering growing interest among researchers because of the shorter composting period and less nitrogen being lost compared to conventional composting [39–41].

Ambient temperature has also been reported to influence the composting period. This effect is more pronounced for composting carried outdoors, e.g., windrow and onsite composting that are exposed to the elements [42–44]. Zhou, et al. [43] studied windrow composting in summer and winter and concluded that the temperature took one day longer to increase during winter. The maximum temperature reached during the thermophilic phase was also lower. Heat loss from the surface of the compost to the environment is also more pronounced in colder temperatures. In large-scale composting operation, uneven temperature distribution may occur due to non-homogeneous mixing or aeration and may end up affecting the compost quality.

#### 2.2.6. pH

The initial pH of the composting materials is influenced by the type of organic wastes. For instance, food waste is slightly acidic, while animal manure is alkaline. Furthermore, the bacterial community in the compost prefers neutral or near-neutral pHs, while the fungal community prefers slightly acidic conditions [45]. Therefore, the optimal pH range varies noticeably for composting, 5.5–8.0 [27,35,46,47]. This explains why pH is not usually adjusted during composting when compared to other biological treatment technologies. However, lower pH values have been shown to influence composting negatively [35,48]. For example, Cao, et al. [35] demonstrated that the initial pH of 5 delayed degradation by seven to ten days and also increased the electrical conductivity of the final mature compost above the acceptable standards (≤4 mS cm^−1^). Such low pHs are characteristic of food waste composting, and some researchers have increased the initial pH of the compost mixture [49].

The pH also varies throughout the composting process. When the process commences, the production of organic acids lowers the pH of the compost. The production of ammonia from the decomposition of nitrogen-containing organic matter increases the pH in the thermophilic stage [33]. NH_4_^+^ and HCO_3_^−^, other decomposition products, act as buffers that maintain the high pH throughout the composting process [35].

### 2.3. The history and current state of the composting process

Composting has a long history that evolved alongside human settlements and the practice of agriculture. Diaz and De Bertoldi [50] present an exhaustive history of composting from the Neolithic period to the 20^th^ century. Research on the composting process and influencing factors can be traced back to the late 1940s and 1950s. Mechanization of composting technology also began during this period, with composting digesters such as the Hardy digester and Dano drums becoming commercially available. The 1960s through to the 1980s witnessed more research on the technical aspects and financial viability of composting facilities, use of compost, the effect of compost on plant growth, and the hygienization aspects of composting [51,52]. Well-known composting technologies such as the aerated static pile were invented during this period [53]. Zheng, et al. [54] refer to this period as the budding stage of composting technology based on the number of patents filed worldwide. The period between 1990 and 2007 was designated as the developing stage and more recent years as the expanding stage. Their comprehensive bibliometric analysis suggests that composting technology research interest has grown steadily over the years, especially with the rise of the sustainable development movement and efforts to minimize wastage and needless pollution.

**Figure 2.**
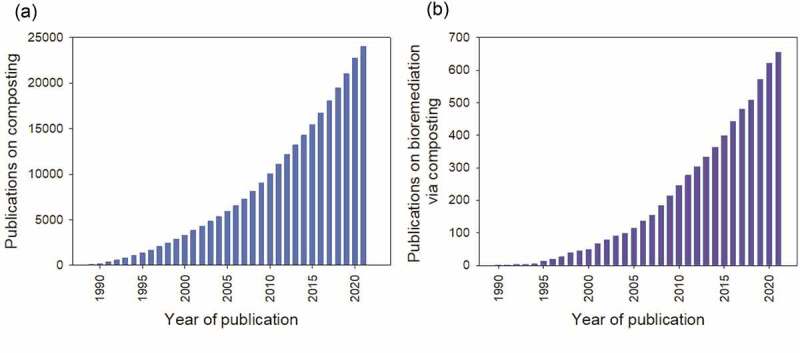
(a) The cumulative number of composting articles (b) bioremediation via composting articles published from 1989 to 2020. The data was sourced from the Web of Science database on August 28^th^, 2020.

Figure 2 represents the cumulative publications on composting covering the years 1989 to 2021. The data was accessed from the Web of Science database and only included Scientific Citation Index Expanded (SCIE) research, review, and early access articles. According to Figure 2(a), a total of 24,000 articles were published in the past three decades. Interest in composting research can also be seen to increase over the period steadily. The research has primarily focused on optimizing and shortening the composting process [55,56], odor control [57,58], microbial community structure [47,59,60], composting application including heat recovery [61–63] and bioremediation [6,9,31]. Figure 2(b) shows the publication of articles on bioremediation in those three decades. Based on the exponential growth, bioremediation research via composting appears to be on the rise. A total of 655 articles have been published between 1990 to 2021, with 40% published in the last six years.

## 3. Bioremediation of recalcitrant organic contaminants

Bioremediation technologies employ microorganisms to degrade organic contaminants. This degradation process can occur via three main pathways: (i) mineralization or metabolism, whereby the microorganisms utilize the contaminant as a nutrient source; (ii) co-metabolism, whereby contaminants that do not serve as a nutrient source are broken down in parallel with metabolic reactions; and (iii) nonspecific oxidation, which involves the extracellular degradation of contaminants [64]. Traditionally, bioremediation has been carried out in various ways, including bioaugmentation and biostimulation. Bioaugmentation involves incorporating specific microbial species capable of mineralizing a contaminant and using it as a nutrient source into a contaminated environment. Biostimulation involves providing rate-limiting nutrients such as phosphorus and nitrogen and supplements such as biosurfactants to microorganisms in a contaminated environment. Bioaugmentation has been conducted using single or multiple strains of bacteria or fungi (mycoremediation).

Bioremediation via composting presents unique advantages over single- or multi-strain bioremediation. Firstly, the composting process comprises several microorganisms that participate in mutualistic, synergistic, and/or competitive relationships. In this diverse microbial structure, a handful of species could completely mineralize or metabolize a contaminant, while other species are only able to co-metabolize or nonspecifically oxidize the contaminants. This makes the composting process robust and highly effective in degrading organic contaminants. In addition, the composting process undergoes certain physicochemical transformations that govern the fate of the organic contaminant in the compost. For example, the high temperatures during the thermophilic stage could cause the volatilization of volatile and semi-volatile compounds. On the other hand, the high temperatures might also increase the bioavailability of certain compounds by making them less viscous. Furthermore, some species can produce biosurfactants that increase the bioavailability of the compounds [65].

This section presents the bioremediation of some common recalcitrant organic pollutants found in the pedosphere and sediment, including polycyclic aromatic hydrocarbons (PAHs), di(2-ethylhexyl) phthalate (DEHP), polychlorinated dibenzo-p-dioxins/furans (PCDD/Fs), total petroleum hydrocarbons (TPH), and pesticides. These compounds are highly hydrophobic and prevalent in the environment as a result of anthropogenic activities. Bioremediation studies, including composting, single- and multi-strain bioremediation, and biostimulation approaches, published in the past ten years, are presented in Tables 2–5.

**Table 2.** Bioremediation and composting of petroleum and petroleum-related contaminants by biological treatment approaches

Biological approaches	Target pollutants	Initial concentration	Size	Period (days)	Reduction (%)	Details	Ref.
Biostimulation and bioaugmentation	TPH	41,065–60,153 mg kg^−1^ of the mixture	2.5–3.0 kg	105	23–40	Contaminated soil mixed with uncontaminated soil in equal parts and molasses, compost, sludge, or activated carbon.	^[104]^
Phytoremediation, biostimulation, and bioaugmentation	TPH	2,500–13,200 mg kg^−1^ of soil	0.5 kg of soil in pots	60	50–62	Bacillus genus (e.g., *Bacillus, Pseudomonas, Listeria, Rothia, Corynebacterium, and Rhodococcus*) with molasses, nutrients, biosurfactants, and/or H_2_O_2_.	^[105]^
Multi-strain mycoremediation	TPH	54,074 mg kg^−1^ of sediment	Lab-scale 100 mL Malt Extract Broth	60	47.6	*Lambertella, Penicillium, Clonostachys*, and *Mucor* supplemented with wood chips. Bacterial species were also said to participate in the degradation	^[4]^
Biosurfactant	TPH	2,642 mg kg^−1^of soil	2.5 kg	28	61.8	Used biosurfactant produced by *Bacillus Malacitensis* isolated from aromatic hydrocarbon-contaminated soil	^[106]^
Composting and phytoremediation	TPH	31,823 mg kg^−1^ of soil	67.5 kg (2:1 mass ratio; soil: compost material)	203 (63 d composting and 140 d phytoremediation)	48	Yard trimmings, cactus, and urea.	^[107]^
Composting	Diesel	26,315 mg kg^−1^ of initial composting material	130 kg	24	90–92	Food waste, sawdust, and mature compost	^[5]^
Composting	PAHs	13.5–15.9 mg kg^−1^ of sewage sludge	0.02 m^3^ closed reactor	39	58.7–76.4	Used Sewage sludge and mushroom residue composting to degrade 6 PAHs	^[1]^
Biostimulation and bioaugmentation with two bacterial strains	PAHs	332 mg kg^−1^ of soil	0.04 m^3^ stainless-steel reactor (1:5 ratio; soil: distilled water)	15	80	Bioaugmented the bioslurry with *Rhodocccus erythropolis* and *Pseudomonas stuzeri*. Biostimulated the process with N, P, and biosurfactants.	^[108]^
Composting	PAHs	6–10 mg kg^−1^of sewage sludge	Commercial-scale windrow (1.5 m wide, 1.2 m high, and 10 m long)	50	62.4–75.2	Sewage sludge, green forest waste, and mature compost.	^[2]^
Composting	PAHs	0.082 mg kg^−1^ sewage sludge	Commercial scale	110	57 ± 31	Dewatered sewage sludge and food industry waste	^[3]^

Remarks: TPH – Total petroleum hydrocarbons; PAHs – Polycyclic aromatic hydrocarbons.

**Table 3.** Bioremediation of phthalate-based plasticizers by biological treatment approaches

Biological approaches	Target pollutants	Initial concentration	Size	Period (days)	Reduction (%)	Details	Ref.
Single-strain bioremediation	3 PAEs (DEHP, DBP, and DnOP)	1,000 mg kg^−1^ of soil	100 g	21	>55	*Rhodococcus* sp. strain WJ4 isolated from soil	^[109]^
Composting	5 PAEs (DEHP, BBP, DBP, DEP, and DMP)	25.5 mg kg^−1^ of sewage sludge	3.6–4.0 kg	60	32.2–78.1	Sewage sludge, rice straw and Pig manure	^[7]^
Composting	DOTP	11,882 mg kg^−1^ of initial compost material mixture	110 kg	35	98	Food waste, sawdust and matured compost	^[6]^

Remarks: DEHP – Di-(2-ethylhexyl) phthalate; DnOP – di-n-octyl phthalate; DMP – dimethyl phthalate; DEP – diethyl phthalate; DBP – di-n-butyl phthalate; BBP – butyl benzyl phthalate; DOTP – dioctyl terephthalate .

**Table 4.** Bioremediation of pesticides by biological treatment approaches

Biological approaches	Target pollutants	Initial concentration	Size	Period (days)	Reduction (%)	Details	Ref.
Multi-strain bioaugmentation	Phorate	100–300 mg kg^−1^ of soil	100 g	42	97.7–98.3	*Brevibacterium frigoritolerans, Bacillus aerophilus and Pseudomonas fulva.*	^[110]^
Phytoremediation + Single-strain bioremediation	Dichlorodiphenyltrichloroethane (DDT)	1.42 mg kg^−1^ of soil	1.5 kg contaminated soil in pots	100	65.6 and 65.9	Tall fescue, perennial ryegrass with biosurfactant-producing *Pseudomonas* sp. SB (10^7^ CFU g^−1^ dry soil per pot).	^[111]^
Multi-strain bioaugmentation	Chlorpyrifos	50 mg kg^−1^ of soil	20 g	10	82	*Pseudomonas, Klebsiella, Stenotrophomonas, Ochrobactrumand Bacillus.*	^[112]^
Composting	Aldrin α-endosulfan β-endosulfan Lindane	0.45–0.65 mg kg^−1^ 1.20–1.40 mg kg^−1^ 0.65 mg kg^−1^ 0.35–0.50 mg kg^−1^of raw material	~100 kg d^−^^[1]^ of waste fed in 3.5 m^3^ continuous rotary drum composting.	100	87.0 86.4 84.0 80.0	Vegetable waste, cow dung, and sawdust.	^[8]^

Remarks: Initial concentrations are per dry weight of soil or initial material mixture.

**Table 5.** Bioremediation and composting of halogenated biphenyls, dioxins, and furans by biological treatment approaches

Biological approaches	Target pollutants	Initial concentration	Size	Period (days)	Reduction (%)	Details	Ref.
Multi-strain bioaugmentation	PCBs	23 mg kg^−1^ of sediment	20 g	21	41–85	Bioaugmentation with *R. ruber, A. xylosoxidans, R. ruber, S. maltophilia, O. anthropic and A. xylosoxidans* isolated from PCB-contaminated sediment.	^[113]^
Composting	PCBs	0.6 mg kg^−1^ of sewage sludge	0.2 m^3^ reactor (1:2; sewage sludge:peat and peat)	124	67	Sewage sludge, bark, and peat	^[11]^
Single-strain mycoremediation	PCDD/Fs	260 ± 37 ng kg^−1^	150 g (dry weight)	60	35 25	*Penicillium Brasilianum* and *Fusarium Solani* (isolated from PCDD/F-contaminated soil) with wood and cardboard chips	^[114]^
Single-strain mycoremediation	PCDD/Fs	6,238 ± 1110 ng I-TEQ kg^−1^ of soil	Carried out in 250 mL glass bottles (1:1; soil:inocula)	72	96	Solid state fermentation coupled with *Pleurotus pulmonarius*	^[115]^
Single-strain mycoremediation	PCDD/Fs	4,432 ± 632 ng WHO-TEQ kg^−1^ of soil	300 g (dry weight) (1:0.5; soil:inocula)	30	60	Solid state fermentation coupled with *Pleurotus pulmonarius*	^[116]^
Composting	PCDD/Fs	16,004 ng-TEQ kg^−1^	85 kg	42	75	Food waste, sawdust, and mature compost	^[10]^
Composting	PCDD/Fs	8,954 ng-TEQ kg^−1^ of soil	89 kg	35	81	Food waste, sawdust, and mature compost	^[9]^

Remarks: PCBs – Polychlorinated biphenyls Octachlorodibenzofuran PCDD/Fs – Polychlorinated dibenzo-p-dioxins/dibenzofurans; I-TEQ – International Toxic Equivalent; WHO-TEQ – World Health Organization Toxic Equivalent.

### 3.1. Bioremediation of petroleum and petroleum-related organic contaminations

Petroleum contaminants are among the most prevalent organic contaminants in the environment because of anthropogenic activities, including petroleum extraction, processing, transportation, storage, and usage. Table 2 presents results from recent studies on the bioremediation of petroleum and petroleum-related contaminants at different scales. Petroleum-related contaminants that have been remediated via bioremediation include diesel, PAH, and TPH. It can be observed that the efficiency in removing petroleum contaminants utilizing biological approaches varies according to the initial concentration, biological approach, and scale. Except for composting, other bioremediation approaches were generally small-scale. This is an advantage of composting in that the scale of remediating TPH can be increased without significantly compromising the removal efficiency. For example, Lin, et al. [5] showed that over 90% of diesel of the initial concentration of 26,315 mg kg^−1^ was degraded via composting for 24 days. This suggests that petroleum products are relatively easier to biodegrade.

Numerous microbial species capable of mineralizing petroleum and petroleum-related compounds have been reported in various studies [12]. These species have been isolated from petroleum-contaminated environments, cultured, and used in bioremediation studies. For example, Abena, et al. [12] identified crude oil-degrading bacterial strains belonging to *Raoultella ornithinolytica, Bacillus subtilis, Serratia marcescens*, and *Acinetobacter lwoffii* species and augmented the strains in contaminated soils, increasing the TPH-degradation to 48.1%. These species release certain enzymes, including alkane hydroxylases and methane monooxygenases, which assist in the breakdown of petroleum. For example, methane monooxygenase can oxidize the C-H bonds of alkanes.

The aerobic bacterial degradation mechanism of petroleum products, especially n-alkanes, is well documented [13,66]. The n-alkanes are broken down into a carbon source for the bacteria through the main pathways: terminal, subterminal, β-, and ω-oxidations. Details of the mechanisms have been extensively presented in review papers on petroleum degradation [66,67]. Briefly, these pathways are catalyzed by monooxygenases to convert them into alcohols. The dehydrogenases catalyze the conversion of the alcohols into aldehydes and ketones, then further into fatty acids. The fatty acids are further oxidized into tricarboxylic acid cycle (TCA) intermediates. On the other hand, the degradation mechanism of aromatic petroleum compounds is more complicated and has been reported to be initiated via oxidative attack with the help of monooxygenases or dioxygenases to produce catechol-like structures before ring cleavage reactions by dioxygenases. The resultant straight chain product goes through the above-mentioned n-alkane oxidation reactions.

Fungal species from genera such as *Aspergillus, Alternaria, Penicillium*, and *Graphium* have been reported to degrade petroleum and petroleum-related compounds [68]. The degradation of complex petroleum-related compounds like PAHs by ligninolytic and non-ligninolytic fungi have been reported in detail by some studies [69,70]. Ligninolytic fungi extracellularly degrade PAHs using lignin-degrading enzymes, including peroxidases and laccase. Both groups of compounds can degrade these compounds intracellularly in reactions mediated by hydrolases and cytochrome P450 monooxygenases. The extracellular degradation produces polar and water-soluble products that can be accessible for fungal and the other microbial metabolisms in that environment. This process can occur during composting, and because of the microbial diversity, there would be a high probability of several species that would be able to metabolize these extracellular degradation products.

Several petroleum-degrading species have been reported in literature, including mesophilic microbes such as *Acinetobacter calcoaceticus, Bacillus simplex, Paenibacillus pabuli, Bacillus pumilus*, and *Pseudomonas aeruginosa*; and thermophilic microbes like *Bacillus megaterium, Aspergillus sp, Pseudoxanthomonas sp., Mucor sp, Rhizopus sp*., and *Shigella flexneri* [13,17,71,72]. This suggests that biodegradation can occur at all stages of the composting process.

### 3.2. Bioremediation of phthalate-based plasticizers

Phthalate-based plasticizers are common plastic and rubber additives that increase flexibility and durability. Consequently, they have become quite prevalent in the environment. Environmentalists and public health experts are greatly concerned about the links of these compounds to endocrine disruption. More toxic phthalates such as diethylhexyl phthalate (DEHP) and di-butyl phthalate (DBP) are already being phased out entirely or in some products, e.g., children’s toys in the EU and the US. Few researchers have also shown interest in using bioremediation techniques to study the effectiveness of removing phthalates, as shown in Table 3. Composting can degrade multi-pollutants, as shown by Fu, et al. [7]. Tran, et al. [6] also showed that pilot-scale food waste composting removed 98% of DOTP with high concentrations of 11,882 mg kg^−1^ after only 35 days of composting. This was significantly higher than the single-strain bioremediation study, which had lower concentrations and a scale over 1,000 times smaller.

The microbial degradation of phthalates involves a series of β-oxidation and de-esterification reactions to produce phthalic, terephthalic, or isophthalic acids [73–76]. Boll, et al. [77] detailed the most updated understanding of microbial degradation of the resultant acids. They reported that almost all the aerobic microorganisms convert these compounds into protocatechuate, a TCA intermediate. These reactions involve three steps for phthalic acid; dioxygenation, dehydrogenation and decarboxylation, and two steps for terephthalic and isophthalic acids; dioxygenation and dehydrogenation. The involved enzymes are decarboxylases, dehydrogenases, and dioxygenases. Several species were found in compost [6,33,78], e.g., *Microbacterium* sp and *Rhodococcus erythropolis, Gordonia sp. Pseudomonas sp. Bacillus sp., Rhizobium sp*., and *Achromobacter sp*. can completely metabolize phthalates, even at high concentrations [73]. Therefore, composting is well equipped to degrade phthalates effectively.

### 3.3. Bioremediation of pesticides

Pesticides, including herbicides, insecticides, and fungicides, have been extensively used in agriculture to boost yield by keeping away pests and weeds. Some of these pesticides from the organochlorine group, e.g., dichlorodiphenyltrichloroethane (DDT), lindane, endrin, dieldrin, endosulfan, and heptachlor, are part of the compounds listed in the Stockholm Convention of POPs and are banned or restricted globally because of the environmental and human health risk. However, these legacy pesticides are highly persistent with incredibly long half-lives and are still found in soil [79] and sediment [80,81]. Table 4 summarizes the results of some recent studies on bioremediation of organochlorine and organophosphate pesticides by biological treatment approaches. Bioremediation of organochlorine (DDT, aldrin, lindane, α- and β-endosulfan) appears slower than organophosphate (phorate and chlorpyrifos) pesticides, which is attributable to their higher toxicity. Egbe, et al. [82] reported that organochlorine pesticides reduced the number of bacterial and fungal species when added to agricultural soils, implying that these compounds are toxic to some microorganisms. Bioremediation also showed high removal efficiency. Ali, et al. [8] reported that composting could simultaneously degrade multiple legacy pesticides and achieve high degradation efficiencies of 80–87% after 100 days of composting.

Some specific strains found in compost can degrade some pesticides. For example, Kumar and Pannu [83] identified *Rhodanobacter lindaniclasticus, Alkaligens faecalis*, and *Pseudomonas aeruginosa* as capable of dechlorinating lindane, Wang, et al. [84] reported that *Stroptomyces* sp. strain can degrade DDT, and Seralathan, et al. [85] confirmed that *Pseudomonas aeruginosa, Ochrobacterium* sp, and *Achromobacter xylosoxidans* degrade endosulfan and use it as a source of sulfur. These organochlorine pesticide-degrading species contain genes, e.g., lin and Esd genes that encode for dehalogenases, hydrolases, dehydrochlorinases, and monooxygenases enzymes that take part in mineralizing these compounds [86]. Hydrolysis, mediated by phosphotriesterase, is the primary step in the bacterial degradation of organophosphate pesticides which causes the cleaving of the P-O/F/S bond separating the two main moieties, which undergo further reactions to produce TCA intermediates [86,87]. Since organophosphates have lower toxicity and less persistence in the environment, many more microbial species documented by Mulla, et al. [87] have been identified to degrade organophosphate pesticides.

### 3.4. Bioremediation of halogenated biphenyls, dioxins, and furans

These persistent organic pollutants (POPs), including polychlorinated dibenzo-p-dioxins and dibenzofurans (PCDD/Fs) and polychlorinated biphenyls (PCBs), stubbornly remain in the soil and sediments and are also carcinogenic and mutagenic. They constitute some of the most toxic compounds known to man and were among the first listed compounds in the Stockholm Convention on POPs. PCBs were manufactured and widely used as coolants for many decades, but like PCDD/Fs, they can also be unintentionally produced during incomplete combustion. Therefore, all combustion sources such as engines, incinerators, and power plants, can produce these compounds. Since they are highly hydrophobic and have a high affinity to particles, they are found mostly in soil and sediments, where they have long half-lives that could last for more than 100 years [88].

Table 5 documents some studies on the bioremediation of these POPs published in the last ten years. The first observation is that the initial concentrations, especially for PCDD/Fs, are much lower than those used in the bioremediation of the other compounds presented in this review. This is probably because of the high toxicity of these compounds. Successful pilot-scale bioremediation of these POPs via composting has been reported [9,10] with removal efficiencies > 75%. Bacterial species with the dioxygenase encoding genes are capable of degrading PCDD/F and PCDD/F-like compounds. Some of these species that have been found in compost include *Sphingomonas sp., Pseudomonas aeruginosa, Acinetobacter sp., Pseudomonas putida, Ralstonia sp., Burkholderia sp., Comamonas testosteroni, Novosphingobium sp., Burkholderia cepacia*, and *Pseudomonas sp*. [9,89,90].

The aerobic bacterial degradation mechanism has been reported to be initiated via either angular or lateral dioxygenation [91]. The degradation mechanisms of these compounds have been carried out using non-chlorinated congeners because they are less stable. The dioxygenation reaction involves the addition of OH to the angular or lateral position. This initial reaction is catalyzed by monooxygenases or hydroxylating dioxygenases. The preceding oxygenation knocks down the planar structure of the molecule in the angular dioxygenation, making the compound less toxic. The series of reactions proceed by opening the aromatic ring producing salicylic acid and catechol, which are further broken down to TCA intermediates. The fungal degradation mechanism involves similar reactions in the degradation of aromatic compounds previously mentioned in Section 3.1.

## 4. Conclusions and future perspectives

From reviewing the recent publications, we can conclude that bioremediation via composting is still a nascent topic but has great potential to remove recalcitrant organic pollutants in soils and sediments. Some of the organic contaminants that have been successfully treated via composting include diesel, total petroleum hydrocarbons (TPH), polycyclic aromatic hydrocarbons (PAHs), polychlorinated biphenyls (PCBs), diethylhexyl phthalate (DEHP), and polychlorinated dibenzo-p-dioxins and dibenzofurans (PCDD/Fs). Generally, highly toxic compounds like PCDD/Fs take longer to degrade, and only lower initial concentrations can be successfully treated. Since composting is a biological process, ensuring that key parameters, including particle size, nutrients, oxygen content, and moisture content, are within the suitable ranges of effective composting enhances the bioremediation. Moisture content of 55–65%, C/N ratio of 25–30 and oxygen content of > 10% in the compost gas have been recommended by researchers as optimal. However, the research on the optimal ranges of the other parameters is still inadequate to draw any certain conclusions. Interesting and important research directions that need further investigation include bio-augmenting/inoculating the composting process with specific microbes capable of degrading a contaminant at different composting stages. Another important topic is studying the pairing of composting with other bioremediation technologies, e.g., using mature compost with residual contamination in mycoremediation or phytoremediation. In addition, investigating the ability of composting to degrade multiple organic contaminants simultaneously is another interesting research area. In conclusion, composting is a suitable bioremediation technology that deserves more attention.

## Highlights

- Composting can successfully bioremediate several recalcitrant organic contaminants
- The microbial diversity of composting offers unique bioremediation merits
- Production of biosurfactants and the high temperatures promote bioavailability
- Ensuring optimal composting conditions enhances the bioremediation
- The exact degradation mechanism of contaminants remains complex and unconfirmed
